# The tumor suppressor miR-138-5p targets PD-L1 in colorectal cancer

**DOI:** 10.18632/oncotarget.9659

**Published:** 2016-05-27

**Authors:** Lian Zhao, Haibo Yu, Shuijing Yi, Xiaowei Peng, Peng Su, Zhiming Xiao, Rui Liu, Anliu Tang, Xiayu Li, Fen Liu, Shourong Shen

**Affiliations:** ^1^ Department of Gastroenterology, The Third Xiangya Hospital, Central South University, Changsha, Hunan, China; ^2^ Hunan Key Laboratory of Nonresolving Inflammation and Cancer, Changsha, Hunan, China; ^3^ Department of Metabolism and Endocrinology, The Second Xiangya Hospital, Central South University, Changsha, Hunan, China; ^4^ Department of Gynaecology and Obstetrics, The Third Xiangya Hospital, Central South University, Changsha, Hunan, China; ^5^ Department of Breast Oncology Plastic and Head and Neck, The Affiliated Cancer Hospital of Xiangya Medical School, Hunan, China

**Keywords:** miR-138-5p, PD-L1, tumor suppressor, colorectal cancer, biomarker

## Abstract

microRNAs (miRNAs) play critical roles in cancer development and progression. This study investigated the effects of miR-138-5p in human colorectal cancer (CRC) development. miR-138-5p was frequently downregulated in CRC tissues and was associated with advanced clinical stage, lymph node metastasis and poor overall survival. We found that miR-138-5p decreased expression of programmed cell death ligand 1 (PD-L1) through interaction with its PD-L1 3′ untranslated region. miR-138-5p also dramatically suppressed CRC cell growth *in vitro* and inhibited tumorigenesis *in vivo*. PD-L1 and miR-138-5p levels were inversely correlated in human CRC tumors, and miR-138-5p inhibited PD-L1 expression in tumor models. These results suggest that miR-138-5p is a tumor suppressor in CRC, and its effects are exerted at least partially through PD-L1 downregulation. Low miR-138-5p and high PD-L1 levels correlated with shorter overall CRC patient survival, indicating that miR-138-5p and PD-L1 may serve as CRC biomarkers for risk group assignment, optimal therapy selection and clinical outcome prediction. Targeting PD-L1, possibly by administering miR-138-5p mimics, might be a clinically effective anti-CRC therapeutic strategy.

## INTRODUCTION

CRC is one of the most common malignancies in the world and is the second leading cause of cancer-related mortality in Western countries [1]. Efforts to elucidate the underlying causes of CRC and to develop more effective therapies have thus far met with only limited success [2]. microRNAs (miRNAs) belong to a class of short, highly conserved non-coding RNAs known to suppress protein coding gene expression through imperfect base pairing with the 3′untranslated regions (UTRs) of target messenger RNAs (mRNAs) [3]. miRNAs have been implicated in the control of various biological processes, such as cell proliferation, apoptosis and differentiation [4–7]. Alterations in miRNA expression have been observed in CRC, and several dysregulated miRNAs, including miR-625-3p [8], miR-99-5b [9], miR-361-5p [10], miR-17-5p [11], miR-137 [12], miR-95 [13], miR-23a [14, 15], miR-155 [16], miR-150 [17], miR-191[18], miR-339-5p [19], miR-429 [20], miR-345 [21], miR-22 [22], miR-638 [23] and miR-138 [24], have been shown to regulate CRC cell growth, apoptosis and metastasis. However, the role of miRNAs in CRC development and progression remains unclear, and more extensive studies are required to identify those miRNAs that may be useful as prognostic predictors and/or therapeutic targets in CRC.

PD-L1 is constitutively activated in tumor cells, promoting tumor survival and growth by increasing the capacity of tumors to evade the immune system [25–31]. Furthermore, PD-L1 was reported as an oncogene that correlates with poor prognosis in several carcinomas [32–35]. In this study, miR-138-5p was shown to be frequently downregulated in CRC tissues and might act as a tumor suppressor. We found that miR-138-5p markedly suppressed CRC cell growth *in vitro* and inhibited tumorigenesis *in vivo* by targeting PD-L1; miR-138-5p expression was inversely correlated with that of PD-L1 in CRC. Low miR-138-5p expression was associated with advanced clinical stage, lymph node metastasis and poor overall patient survival, and high PD-L1 expression correlated with decreased overall patient survival.

## RESULTS

### miR-138-5p is frequently downregulated in CRC

**Figure 1:**
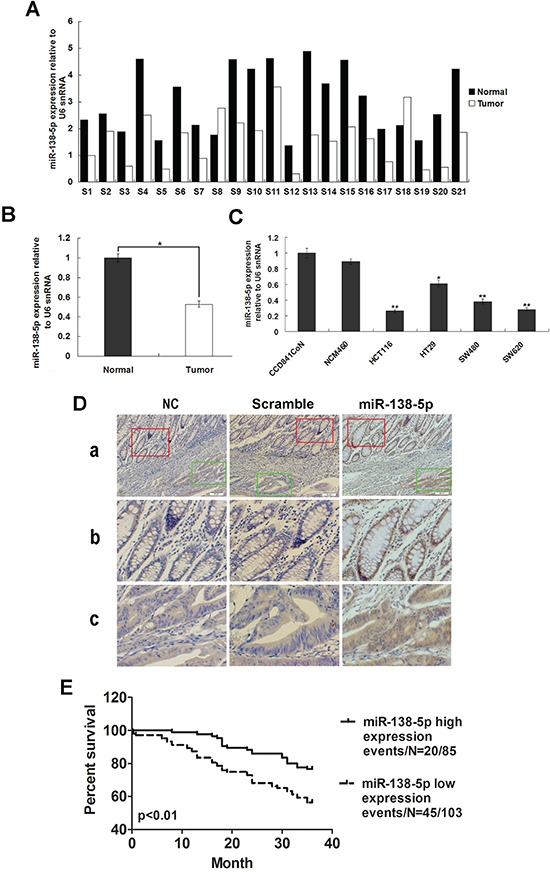
miR-138-5p is frequently downregulated in CRC miR-138-5p expression in 21 CRC tissues and corresponding adjacent normal tissues as determined by qRT-PCR analysis **A.** U6 was the endogenous control. Relative miR-138-5p expression in CRC tissues **B.** miR-138-5p expression in two normal epithelial cell lines and four colon cancer cell lines as determined by qPCR (**P<0.01) **C.** U6 was the endogenous control. Data are presented as the mean ± SD from at least three separate experiments. Detection of miR-138-5p by ISH in CRC and corresponding adjacent normal tissues **D.** Tissue samples were cut into serial paraffin sections. “Scramble” and “NC, without probe” were used as controls. Row b shows magnified images of staining in adjacent normal tissues (red box in row a). Row c shows magnified images of staining in tumor tissues (green box in row a). Magnifications: ×100 (a) and ×200 (b and c). Kaplan-Meier analysis indicated that miR-138-5p downregulation was associated with poorer overall CRC patient survival (*P*=0.001) **E.**

miR-138-5p expression was measured in 21 CRC samples and corresponding adjacent normal tissues by qRT-PCR. miR-138-5p downregulation was detected in 19/21 (90.48%) of CRC tumors (Figure 1A). Average miR-138-5p expression was approximately 2.3-fold lower in CRC specimens as compared with corresponding adjacent normal tissues (*P*<0.05, Figure 1B). miR-138-5p expression was also downregulated in CRC cell lines compared with normal colonic epithelium cell lines (Figure 1C). In situ hybridization (ISH) analysis in 188 CRC samples showed miR-138-5p downregulation in 92/188 (48.9%) tumors (Figure 1D). The clinical association analysis found that low miR-138-5p expression was correlated with advanced clinical stage (*P*<0.05) and lymph node metastasis (*P*<0.05, Table 1). Kaplan-Meier analysis indicated that low miR-138-5p expression was associated with poorer overall survival (log-rank test, *P*=0.001, Figure 1E). Further, multivariate Cox regression analysis revealed that low miR-138-5p expression is an independent prognostic factor for poor survival of CRC patients (*P*<0.01, Table 2).

**Table 1:** Correlations between clinicopathological features and miR-138-5p expression in CRC

Feature	All	miR-138-5p level		
		Low (n)	High (n)	*P*-value
PD-L1 expression				
PD-L1 high	98	73(74.5%)	25(25.5%)	
PD-L1 low	90	30(33.3%)	60(66.7%)	0.000*
age				
<55	87	47(54.0%)	40(46.0%)	0.884
≥55	101	56(55.4%)	45(44.6%)	
sex				
Male	120	67(55.8%)	53(44.2%)	0.702
Female	68	36(52.9%)	32(47.1%)	
Differentiation				
Well	61	34(55.7%)	27(44.3%)	0.855
Moderate	98	52(53.1%)	46(46.9%)	
Poor	29	17(58.6%)	12(41.4%)	
T status				
T1	9	9(8.7%)	0(0.0%)	0.008*
T2	14	11(10.7%)	3(3.5%)	
T3	153	77(74.8%)	76(89.4%)	
T4	12	6(5.8%)	6(7.1%)	
TNM stage				
I	20	18(90%)	2(10%)	0.000*
II	78	31(39.7%)	47(60.3%)	
III	60	33(55%)	27(45%)	
IV	30	21(70%)	9(30%)	
Dukes stage				
A stage	30	21(70%)	9(30%)	0.022*
B stage	66	27(40.9%)	39(59.1%)	
C stage	57	32(56.1%)	25(43.9%)	
D stage	35	23(65.7%)	12(34.3%)	
Lymph node metastasis				
N0	98	39(37.9%)	59(69.4%)	0.000*
N1	90	64(62.1%)	26(30.6%)	
M status				
M0	158	82(79.6%)	76(89.4%)	0.068
M1	30	21(20.4%)	9(10.6%)	

* *P*<0.05.

**Table 2:** Cox regression analysis of prognostic factors for overall CRC patient survival (n=188)

Clinicopathological features	Univariate Analysis		Multivariate Analysis	
	HR (95%Cl)	*P*-value	HR (95%Cl)	*P*-value
miR-138-5p downregulation	0.466(0.274-0.790)	0.001*	0.493(0.273-0.890)	0.019*
Gender	0.688(0.402-1.176)	0.535		
Age	0.677(0.465-1.241)	0.688		
T status	1.605(0.969-2.658)	0.066	1.561(0.951-2.564)	0.078
Lymph node metastasis	1.990(1.393-2.841)	<0.001*	1.831(1.296-2.587)	0.001*
M status	2.819(1.632-4.869)	<0.001*	2.65(2.015-4.542)	<0.001*
TNM stage	2.623(1.959-3.512)	<0.001*	2.004(1.384-2.901)	<0.001*
Dukes stage	2.793(2.093-3.728)	<0.001*	2.113(1.492-2.992)	<0.001*

CI = confidence interval; HR = hazard ratio

* *P*<0.05.

### PD-L1 is a direct miR-138-5p target

**Figure 2:**
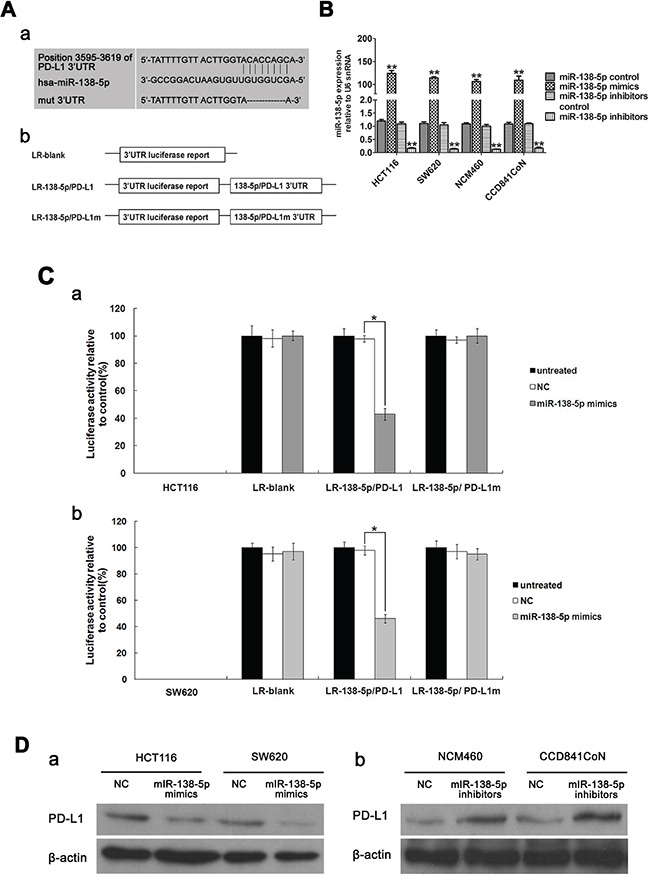
PD-L1 is a direct target of miR-138-5p in CRC cells Base pairing between miR-138-5p and PD-L1 was predicted by MIRBASE software. Schematic representation of the three reporter constructs: LR-blank, LR-138-5p/PD-L1 and LR-138-5p/PD-L1m (where “m” indicates the mutant construct) **Aa.** LR, luciferase reporter **Ab.** miR-138-5p expression was detected via qPCR in HCT116, SW620, NCM460 and CCD841CoN cells transfected with miR-138-5p mimics and inhibitors (**P<0.01). **B.** Data were confirmed in three experiments. Each of the luciferase reporter plasmids was cotransfected as indicated (untreated; NC, scramble; miR-138-5p mimic) into HCT116 **Ca.** and SW620 **Cb.** cells. Luciferase activity was measured after 36 h, and transfection efficiency differences were normalized to renal activity (*P<0.05). Data were confirmed in three experiments. PD-L1 was detected via western blotting in HCT116 and SW620 cells transfected for 48 h with miR-138-5p mimics or NC oligos (miR-138-5p mimics control) **Da.** PD-L1 was detected in NCM460 and CCD841CoN cells transfected for 48 h with miR-138-5p inhibitors or NC oligos (miR-138-5p inhibitors control) **Db.**

To clarify the relationship between miR-138-5p and PD-L1, basic information about hsa-miR-138-5p was collected from miRBase. PD-L1 is a putative miR-138-5p target predicted by MIRDB (Figure 2A). To verify PD-L1 targeting by miR-138-5p, reporter constructs in which the PD-L1 3′UTR, either wild type or mutated in the miR-138-5p binding sites, was cloned downstream of the luciferase open reading frame (Figure 2Ab). miR-138-5p mimic and inhibitors were transfected into HCT116, SW620, NCM460 and CCD841CoN cells (Figure 2B). When the PD-L1 3′ UTR was attached to the luciferase gene, luciferase activity decreased significantly (*P*<0.05) in HCT116 and SW620 cells transfected with miR-138-5p mimics, demonstrating that PD-L1 was the target of miR-138-5p (Figure 2C). Furthermore, expression of mutant PD-L1 3′ UTR restored luciferase activity. To examine the effect of miR-138-5p on endogenous PD-L1 expression, two cell lines with low miR-138-5p expression, HCT116 and SW620, were transfected with miR-138-5p mimics; PD-L1 protein levels were decreased (Figure 2Da). When two cell lines with high miR-138-5p expression, NCM460 and CCD841CoN, were transfected with miR-138-5p inhibitors, PD-L1 levels were increased (Figure 2Db).

### miR-138-5p inhibits cell growth and blocks S-phase entry partially through PD-L1 downregulation *in vitro*

**Figure 3:**
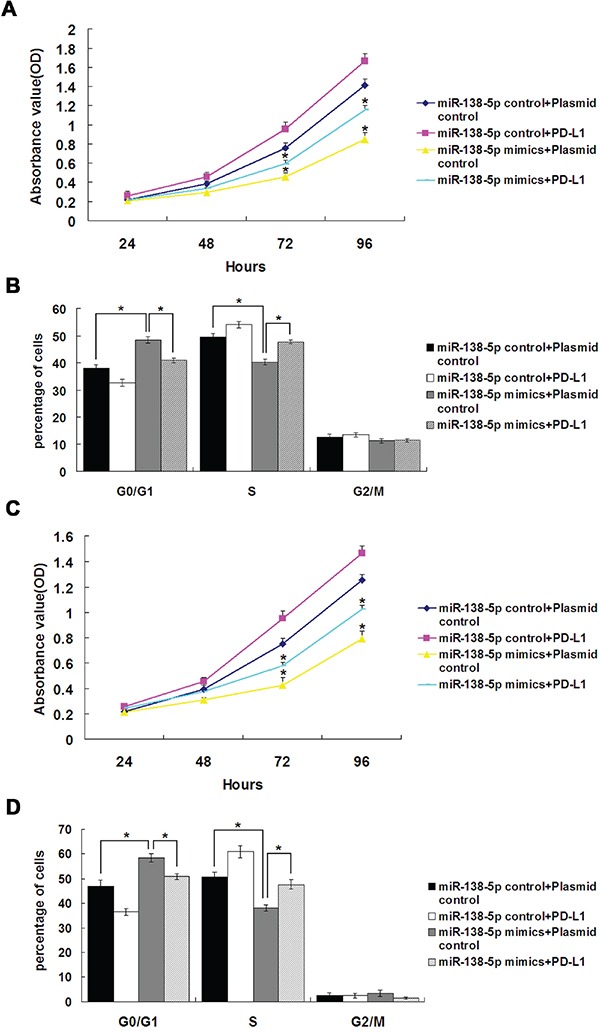
PD-L1 overexpression reversed the effects of miR-138-5p on cell cycle distribution and proliferation in CRC cell lines HCT116 cell proliferation **A.** and cell cycle distribution **B.** after cotransfection with miR-138-5p and PD-L1 or control plasmids. SW620 cell proliferation **C.** and cell cycle distribution **D.** after cotransfection with miR-138-5p and PD-L1 or control plasmids. Data were confirmed in three experiments (*P<0.05).

miR-138-5p was ectopically expressed in CRC cell lines. We determined the effects of miR-138-5p overexpression or inhibition on cell proliferation via MTT assay. HCT116 and SW620 cells (which have low endogenous miR-138-5p expression) transfected with miR-138-5p mimics showed decreased proliferation (*P*<0.05), which was rescued by overexpression of the target gene, PD-L1 (Figure 3A & 3C). In HCT116 and SW620 cells transfected with miR-138-5p mimics, the number of cells in S phase of the cell cycle decreased and the number in G1 phase increased (*P*<0.05, Figure 3B & 3D), and this was again rescued by PD-L1 overexpression (*P*<0.05).

**Figure 4:**
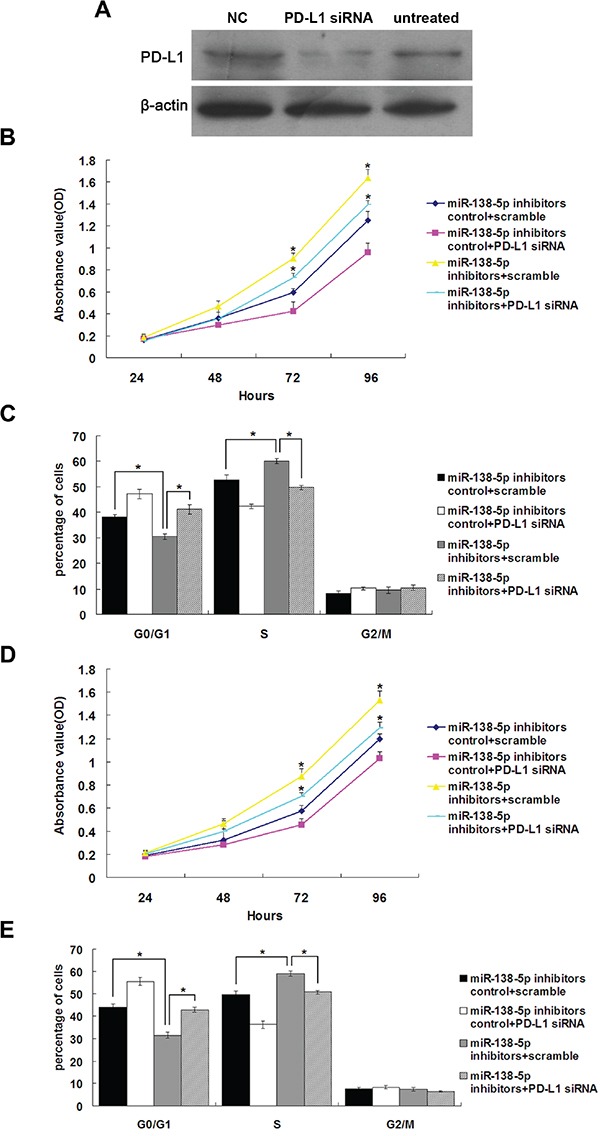
The growth-promoting effects of miR-138-5p inhibition were attenuated by endogenous PD-L1 knockdown in colonic epithelium cells Western blot analysis of PD-L1 in NCM460 cells 48 h post-transfection with PD-L1 siRNA **A.** “NC, scramble” and untreated were used as controls. NCM460 cell proliferation **B.** and cell cycle progression **C.** after cotransfection with the miR-138-5p inhibitor and PD-L1 siRNA or scramble. CCD841CoN cell proliferation **D.** and cell cycle distribution **E.** after cotransfection with the miR-138-5p inhibitor and PD-L1 siRNA or scramble. Data were confirmed in three experiments (*P<0.05).

PD-L1 small interfering RNA (siRNA) transfected into NCM460 cells reduced PD-L1 protein levels, while the negative control (scramble) had no effect (Figure 4A). NCM460 and CCD841CoN cells (with high endogenous miR-138-5p expression) transfected with miR-138-5p inhibitors showed increased proliferation (*P*<0.05), and this was rescued by PD-L1 siRNA transfection (Figure 4B & 4D). In NCM460 and CCD841CoN cells transfected with miR-138-5p inhibitors, the number of cells in S phase of the cell cycle increased and the number in G1 phase decreased (*P*<0.05, Figure 4C & 4E); this was rescued by PD-L1 siRNA transfection (*P*<0.05). Collectively, these results suggest that miR-138-5p expression in CRC cells is important for both proliferation and cell cycle progression, and miR-138-5p acts by downregulating PD-L1.

### Ectopic miR-138-5p expression suppresses CRC cell tumorigenicity *in vivo*

**Figure 5:**
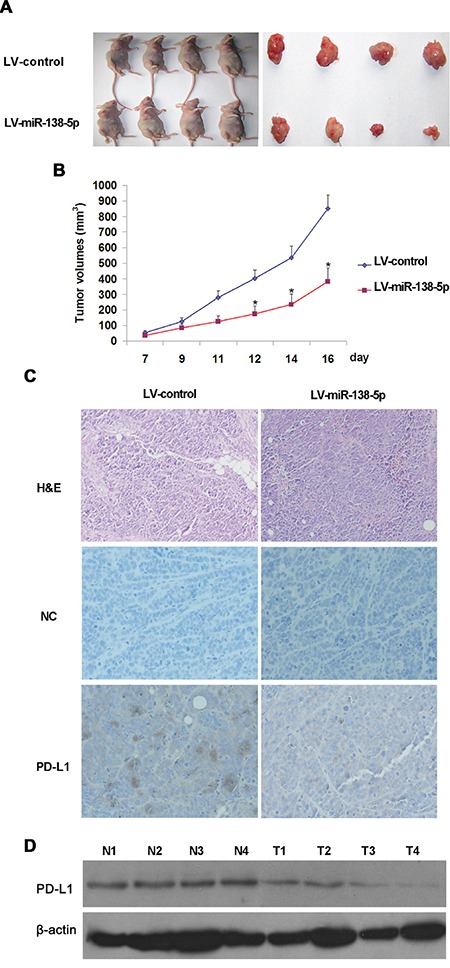
miR-138-5p suppressed SW620 cell tumor growth *in vivo* SW620 cells were infected with LV-miR-138-5p or LV-control, and six million cells in 0.2 ml of growth medium were subcutaneously injected into BALB/c nude mice (four in each group). Tumor formation in SW620-LV-miR-138-5p or SW620-LV-control nude mice **A.** Tumor volume was significantly lower in SW620-LV-miR-138-5p mice as compared with SW620-LV-control mice (*P<0.05) **B.** Data is presented as the mean ± standard deviation (SD) of four mice. PD-L1-stained SW260-LV-miR-138-5p and SW620-LV-control tumors 16 d after transplantation (magnification: ×200) **C.** PD-L1 detected in tumors via western blotting 16 d after transplantation **D.** N1–N4 were derived from SW620-LV-control mice and T1–T4 were from SW620-LV-miR-138-5p mice.

Two stable cell lines derived from SW620, SW620-miR-138-5p and SW620-scramble, were established using LV-miR138-5p and LV-scramble, respectively. SW620-miR-138-5p and SW620-scramble cells were inoculated into mice (four in each group). SW620-miR-138-5p cells exhibited significantly reduced tumor growth compared with SW620-scramble cells (Figure 5A&5B). On day 12, average tumor size in SW620-miR-138-5p mice was reduced as compared to controls (*P*<0.05). Immunohistochemical (IHC) (Figure 5C) and western blot analyses (Figure 5D) revealed that PD-L1 expression was lower in tumors derived from SW620-miR-138-5p mice as compared to controls. These studies demonstrated that miR-138-5p downregulated PD-L1 and inhibited CRC cell tumorigenicity in nude mice.

### miR-138-5p and PD-L1 levels are negatively correlated in CRC

**Figure 6:**
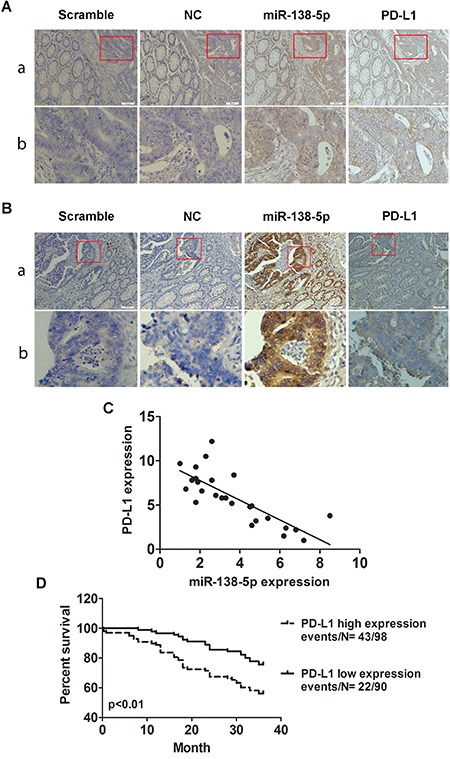
Correlation between miR-138-5p and PD-L1 expression in human CRCs ISH detection of miR-138-5p and IHC detection of PD-L1 in CRCs with low **A.** or high **B.** miR-138-5p expression tissue samples which were cut into serial paraffin sections. “Scramble, miR-138-5p negative control sequence” was the ISH negative control and “NC” was the IHC negative control. Row b shows magnified tumor tissue images (red box in row a). Magnifications: ×100 (a) and ×200 (b). miR-138-5p and PD-L1 mRNA levels were inversely correlated in CRC tissue samples (two-tailed pearson's correlation analysis, r=−0.785; *P*<0.01) **C.** Kaplan-Meier analysis indicated that PD-L1 upregulation was associated with poorer overall patient survival (P=0.0024) **D.**

We assessed PD-L1 and miR-138-5p levels in CRC tissues using ISH, IHC and qRT-PCR. We observed an inverse correlation between PD-L1 and miR-138-5p levels in tumor tissues by all methods (Figure 6A–6C). Patients with high PD-L1 expression had an increased risk of death (*P*=0.0024, Figure 6D), indicating that PD-L1 expression could be a prognostic factor for CRC.

## DISCUSSION

miRNAs, such as oncomiRs or anti-oncomiRs, play critical roles in the initiation and progression of human cancers through post-transcriptional regulation of gene expression [36]. Previous studies indicated that miR-138 might be a tumor suppressor in some cancers, including colorectal cancer, ovarian cancer, head and neck squamous cell carcinoma, nasopharyngeal carcinoma and pancreatic cancer [8, 24, 37-41]. However, the functional role and mechanistic action of miR-138-5p in CRC remained largely unclear. Our study showed miR-138-5p was downregulated in CRC tumor samples as compared with corresponding adjacent normal tissues (Figure 1). We examined the effects of miR-138-5p on CRC cells *in vitro* and *in vivo*. Our results indicated that miR-138-5p reduced CRC cell proliferation and blocked the G1/S transition (Figure 3). In addition, we demonstrated that miR-138-5p overexpression suppressed tumor growth *in vivo* (Figure 5).

PD-L1 expression has been studied in different cancers including melanoma and cancers of the kidney, lung, pancreas, esophagus, ovary, colorectal, breast, head and neck, and was correlated with clinicopathological tumor features in several studies [42–49]. Recent clinical trials demonstrated that blocking the PD-1/PD-L1 pathway induces durable remission in patients with advanced solid tumors. Colorectal cancer appeared to respond poorly to PD-1 or PD-L1 antibody blockade in a clinical trial [50]. Several other factors may be involved in CRC patient survival time [51, 52], and the role of PD-L1 in CRC cells must be further investigated.

miRNAs are known to perform their biological functions by downregulating expression of their target genes. The predicted miR-138-5p binding site was present in PD-L1 3′ UTRs. Whether PD-L1 is the only direct target of miR-138-5p is still unknown. Luciferase activity assay results verified that PD-L1 was a target of miR-138-5p. PD-L1 levels were decreased by ectopic miR-138-5p expression in CRC cells (Figure 2Da). The present study showed that PD-L1 overexpression in CRC cells can rescue them from miR-138-5p-induced cell cycle arrest and reduced proliferation (Figure 3). The tumor suppressive role of miR-138-5p in CRC is thus at least partly realized by downregulating PD-L1. A previous study demonstrated that PD-L1 knockdown by siRNA reduced cell proliferation *in vitro* similarly to miR-138 restoration [33]. We demonstrated a direct link between miR-138-5p and PD-L1 expression in CRC patients, and observed that PD-L1 and miR-138-5p levels were inversely correlated in human CRC specimens (Figure 6C). We also showed that low miR-138-5p expression correlated with advanced clinical stage and lymph node metastasis. Importantly, low miR-138-5p (Figure 1D) and high PD-L1 levels (Figure 5D) were correlated with shorter overall CRC patient survival, indicating that miR-138-5p and PD-L1 may serve as CRC biomarkers for risk group assignment, optimal therapy selection and clinical outcome prediction. Targeting PD-L1, possibly by administering miR-138-5p mimics, might be a clinically effective anti-CRC therapeutic strategy.

## MATERIALS AND METHODS

### Cell lines, plasmids and miRNAs

The human CRC cell lines HCT116, SW620, SW480 and HT29 and human normal colonic epithelium cell lines NCM460 and CCD841CoN were obtained from the Chinese Academy Medical Science (Beijing, China). CRC cell lines were cultured in RPMI-1640 medium containing 10% fetal bovine serum (FBS). NCM460 and CCD841CoN cell lines were cultured in DMEM medium containing 15% FBS. Cells were cultured at 37°C with 5% CO_2_. miRNA mimics, inhibitors and negative control sequences were synthesized and purified by Shanghai Gene Pharma Co (Shanghai, China). The PD-L1 expression plasmid was obtained from OriGene (Beijing, China).

### Tumor samples

CRC patient tumor samples were collected from the Xiangya First, Second, and Third Hospitals of Central South University. Written informed consent was obtained from all study participants. Tissue sample collection and use protocols were approved by the ethical review committees of the Xiangya Hospital Ethic Committee of Central South University.

### Quantitative real-time PCR

Total RNA was isolated from CRC surgical specimens and reverse transcribed as previously described [53]. Real-time quantitative PCR was performed using an IQ5 Multicolor Detection System (Bio-Rad) with a Hairpin-it miRNAs qPCR Quantification Kit (GenePharma, Shanghai, China) according to the manufacturer's instructions. The primers used for miRNA detection by qRT-PCR were designed based on sequences provided by the Sanger Center miRNA Registry. The U6 snRNA was used as an endogenous control. The following program was used for the RT reaction: 16°C for 30 min, 42°C for 30 min and 85°C for 10 min. The following program was used for qPCR: 95°C for 3 min, 40 cycles of 95°C for 12 s and then 62°C for 35 s. The primers used were PD-L1 (forward, 5′-CATCTTATTATGCCTTGGTGTAGCA-3′; reverse, 5′- GGATTACGTCTCCTCCAAATGTG-3′) and β-actin (forward, 5′-GCATCCCCCAAAGTTCACAA-3′; reverse, 5′-AGGACTGGGCCATTCTCCTT-3′). The following program was used for PCR: 94°C for 5 min, 35 cycles of 94°C for 30 s, 58°C for 40 s and 72°C for 30 s, and then 72°C for 10 min. Relative expression changes were calculated using the 2^−ΔΔCT^ (where CT is threshold cycle) method. Three parallel repeats were performed for each sample in each experiment and results were expressed as the mean of three independent experiments.

### Cell proliferation assay

CRC cells were transfected with miR-138-5p mimics or inhibitors for 24 h, and proliferation was examined. Briefly, 2000 cells from each group (untreated, miR-138-5p mimics, miR-138p inhibitors and negative control) were plated in each well of five 96-well plates in 200μL of medium. Cells were treated with 20μL of 5mg/ml MTT (tetrazoliumsalt 3-[4,5-dimethylthiazole-2-yl]-2,5-diphenyltetrazolium bromide) in complete medium for 4 h, and were then dissolved in dimethylsulfoxide (DMSO). Absorbance at 490 nm was measured using an enzyme-linked immunosorbent assay (ELISA) plate reader (Sunrise remote, Tecan Austria GmbH). One plate was analyzed immediately after cells adhered (approximately 4 h after plating). One plate per day was examined for the next four days. Each experiment was repeated three times independently.

### Flow cytometry analysis

Cells were washed twice with phosphate-buffered saline (PBS) and fixed in 70% ethanol overnight. Then, cells were centrifuged at 1,200×g for 5 min, resuspended in 50μg/ml propidium iodide (Sigma-Aldrich) in PBS and immediately analyzed by flow cytometry using a MOFLO XDP flow cytometer (Beckman Coulter, Fullerton, CA). The appropriate forward and side scatter gates were used, and 1×10^5^ cells were examined per experiment. Data were analyzed with Modfit software (Verity Software House, Topsham, ME) and values were expressed as the mean and standard deviation of three independent experiments.

### siRNAs and western blotting

The PD-L1-specific siRNA sequence (GenBank Accession No.NM_014143) [54] was 5′-GATATTTGCTGTCTTTATA-3′. PD-L1 siRNA and scramble sequences were synthesized and purified by Shanghai Gene-Pharma Co. (Shanghai, China) and transfected into cells using Lipofectamine 2000 reagent (Invitrogen, USA) according to the manufacturer's instructions.

Western blotting was performed as described previously [53]. The β-actin antibody was purchased from Sigma-Aldrich (St.Louis, MO) and the PD-L1 antibody was obtained from Millipore (Cat.ABC324).

### In situ hybridization and immunohistochemistry

ISH was used to detect miR-138-5p expression in CRC tissue samples. Oligonucleotide probes complementary to hsa-miR-138-5p (where hsa indicates Homo sapiens) (product 38511-01; Exiqon) and scramble (normal control) were digoxigenin labeled at the 5′ terminal end. Tumor and corresponding adjacent normal tissues were formalin fixed, paraffin embedded and sectioned. Probes were detected using an enhanced sensitive ISH detection kit (Boster Inc., Wuhan, China) according to the manufacturer's instructions. Negative controls did not include probes. MiR-138-5p staining was scored according to intensity and proportion: 0–1 (0–25%), 1–2 (26–50%), 2–3 (51–75%), and 3–4 (76–100%). The sum of intensity and percentage counts was used as the final score. Expression scores greater than or equal to 2 were defined as high, whereas scores lower than 2 were indicative of low expression. The detailed method was applied as previously described [55].

To examine PD-L1 expression in tissues via IHC, sectioned specimens were deparaffinized and dehydrated. Samples that were not exposed to the primary antibody were used as negative controls. The scoring system used in grading PD-L1 expression was described previously [56]. Tumors with strong or moderate immunostaining intensity were classified as having positive (+) expression, whereas tumors with absent or weak immunostaining were classified as having negative (−) expression. miR-138-5p and PD-L1 staining were scored independently by two pathologists blinded to patient clinical characteristics.

### Luciferase reporter assay

Basic information about hsa-miR-138-5p was collected from miRBase (http://microrna.sanger.ac.uk/sequences). The sequence and the possible targets were predicted by the online software MIRDB (http://mirdb.org/cgi-bin/search.cgi?searchType=miRNA&full=mirbase&searchBox=MIMAT0000430). Two single strands of the target gene 3′ UTR containing the miRNA binding sites were synthesized (accession number NM_014143 for PD-L1). Two single strands of the target gene 3′ UTR with 7 bases deleted in the miR-138-5p binding site were synthesized as mutant controls. HindIII and SpeI restriction sites were inserted at both ends of the oligonucleotides for further cloning. The following oligonucleotides were used in this study: the 3′ UTR of PD-L1 (sense, 5′- CTAGTAAATATTCTTATTTATTTTGTTACTTGGTACACCAGCATGTCCATTTTCTTGTTTATTTTA-3′; antisense, 5′- AGCTTAAAATAAACAAGAAAATGGACATGCTGGTGTACCAAGTAACAAAATAAATAAGAATATTTA-3′) and the 3′ UTR of mutant PD-L1 (sense, 5′- CTAGTAAATATTCTTATTTATTTTGTTACTTGGTA-------ATGTCCATTTTCTTGTTTATTTTA-3′; antisense, 5′-AGCTTAAAATAAACAAGAAAATGGACAT-------TACCAAGTAACAAAATAAATAAGAATATTTA-3′; dashes indicate CACCAGC deletion). Sense and antisense strands were annealed, digested with HindIII and SpeI, and ligated into the pmir-Report luciferase vector (Ambion, Austin, TX). Three luciferase reporters (LRs) were constructed: LR-blank (no insertion), LR-138-5p/PD-L1 and LR-138-5p/PD-L1m (where m indicates mutant). Cells were seeded into 24-well plates and 300ng of reporter plasmid, 30ng of renal plasmid and 20nmol of miR-138-5p mimic were cotransfected into cells using Attractene Transfection Reagent. Cell lysates were collected 36 h post-transfection and assayed for luciferase activity using a luciferase assay kit (Promega, Madison, WI). Renal activity was measured using an enzyme assay kit (Promega) and results were normalized to renal activity.

### Experiments in nude mice

Four-week-old specific-pathogen-free (SPF) BALB/c nude mice were purchased from SLAC Laboratory Animal Co., Ltd. (Shanghai, China). All animal procedures were performed according to approved protocols and in accordance with recommendations for the proper use and care of laboratory animals. A xenograft model was used to examine the effects of miR-138-5p on tumor growth in CRC cells *in vivo*. SW-620 cells were infected with LV-miR138-5p or LV-scramble, and six million cells in 0.2 ml of growth medium were subcutaneously injected into BALB/c nude mice. The first tumor was detected one week after inoculation. Mice were killed 9 d after the initial tumor detection, and tumors were harvested, weighed and snap frozen.

### Statistical analysis

The differences between groups were tested examined using the Student's t test or a one-way analysis of variance (ANOVA) test. Survival analysis was performed using Kaplan–Meier plots and log-rank tests. Correlations between miR-138-5p expression and clinicopathological characteristics were analyzed by Pearson's χ^2^ test and Fisher's exact test. Univariate and multivariate Cox proportional hazard regression models were used to assess survival hazard. Statistical analyses were performed using SPSS v16.0. Differences were considered significant when *P*<0.05.
